# Giant myxopapillary ependymoma of the lumbosacral spine

**DOI:** 10.11604/pamj.2021.39.164.29765

**Published:** 2021-07-01

**Authors:** Inas El Kacemi, Gazzaz Miloudi

**Affiliations:** 1 Service de Neurochirurgie, Hôpital Militaire d’Instruction Mohammed V, Faculté de Médecine et de Pharmacie, Université Mohammed V de Rabat, Rabat, Maroc

**Keywords:** Lumbosacral spine, myxopapillary, geant ependymoma

## Image in medicine

Myxopapillary ependymomas rarely present as a primary intralumbosacral lesion, and extensive sacral osteolysis is unusual. We report a case of a 57-year-old man presented with a 10-year history of low back pain. Two months before presentation, he had bladder dysfunction. On examination, there was a reduced range of movement of the lumbar spine without other neurologic deficits. Computed tomographic images of the lumbosacral spine showed lysis of whole sacrum. Magnetic resonance imaging demonstrated a giant lumbosacral mass. Because of the extent of bone destruction, only decompression and subtotal removal of the tumor could be performed, and the patient was referred for local radiotherapy. Histological findings from the tissue removed during surgery showed myxopapillary ependymoma with a positive margin. He had no low back pain at the last follow-up examination and has resumed full activities with normal bowel, bladder, and sexual function.

**Figure 1:**
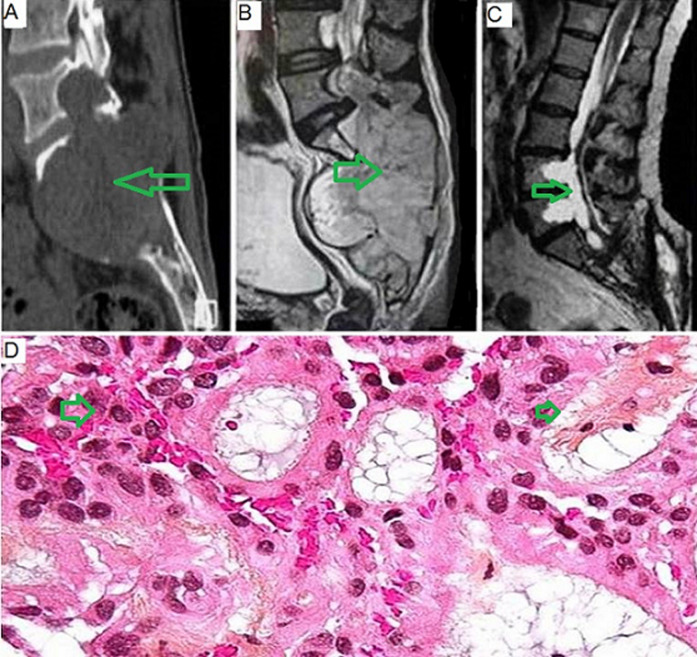
A) sagittal computed tomographic images showing extent of bone destruction; B) sagittal T2 magnetic resonance imaging (MRI) showed hyperintense lesion extending through L3-L4 disc into the whole of sacrum, and this tumor had endopelvic extension and push the rectum and the bladder forwardly; C) postoperative sagittal T2 MRI showed subtotal removal of the tumor; D) histological examination of the tissue removed during the surgery showed that the myxoid area was positioned between small vessels, around which small tumor cells aggregated. The tumor cells were positive for glial fibrillary acidic protein; (top) hematoxylin and eosin, x100 and (bottom) glial fibrillary acidic protein, x100

